# Facile Synthesis of Nd_2_Fe_14_B Hard Magnetic Particles with Microwave-Assisted Hydrothermal Method

**DOI:** 10.3390/molecules28237918

**Published:** 2023-12-03

**Authors:** Ling Wang, Xiaofen Xu

**Affiliations:** Department of Chemistry and Chemical Engineering, Lyuliang University, Lvliang 033000, China

**Keywords:** microwave-assisted hydrothermal method, Nd_2_Fe_14_B, coercivity

## Abstract

The synthesis of Nd-Fe-B magnetic powders via chemical techniques presents significant promise, but poses challenges due to their inherent chemical instability. In this investigation, Nd-Fe-B hard magnetic particles were synthesized utilizing an eco-friendly and simple microwave-assisted hydrothermal synthetic method. The technique involves the synthesis of the Nd-Fe-B oxide precursor using the microwave-assisted hydrothermal method, followed by reduction–diffusion using CaH_2_. The microwave-assisted hydrothermal technique presents a viable approach for preparing Nd-Fe-B precursor particles, offering advantages such as time and energy efficiency and environmental sustainability. The synthesized Nd-Fe-B particles demonstrated a coercivity of up to 2.3 kOe. These magnetic particles hold significant potential for use in high-performance permanent magnets, and can effectively contribute to developing high-energy density exchange-coupled nanocomposite magnets. This study also offers valuable insights into the design and synthesis of additional magnetic materials based on rare earth elements.

## 1. Introduction

Rare earth element-based permanent magnets—Nd_2_Fe_14_B magnets in particular— have found extensive applications across various industries, including transportation, information technology, aerospace, energy, machinery, and others on account of their exceptional magnetic characteristics. The rapid progression of associated application domains, especially in the green energy sector, has increased demand for improved magnetic characteristics and temperature stability [1,2].

Nd-Fe-B-sintered magnets are fabricated using the powder metallurgy technique, which involves multiple preparation procedures. However, this method faces challenges, particularly in producing magnetic powder, which serves as a bottleneck and hinders the advancement of magnet performance in these operations. Currently, the approach yields magnetic powder with a wide particle size range and low morphological uniformity [3,4]. Considering that the coercivity of magnets is a function of their structure, it is evident that the particle size distribution and morphology of magnetic particles are closely linked to the microstructure of post-sintered magnets. Therefore, improving the powder production process emerges as a viable approach to enhance the magnetic properties of magnets. Hence, the utilization of a chemical method for the preparation of Nd_2_Fe_14_B has recently emerged as an attractive subject of investigation, mostly due to its inherent simplicity. In particular, wet-chemistry techniques can generate a diverse range of particle sizes, spanning from nanometers to micrometers, by manipulating the interplay between growth and nucleation processes. Wet chemistry provides several additional advantages, including lower energy consumption, simplified equipment requirements, convenient alloying capabilities, reduced raw material expenses, and the potential for scalability [5,6,7,8].

The chemical production of Nd_2_Fe_14_B particles has attracted enormous scientific attention recently. The preparation of Nd_2_Fe_14_B by using the sol-gel method had been attempted [9,10,11,12]. In 2010, Deheri et al. fabricated Nd-Fe-B magnetic particles with an average particle size of ~50 nm by employing the sol-gel method of chloride base metal salts [11]. The thermal stable sol was prepared by adding a metal salt solution into a citric acid aqueous solution and a glycol aqueous solution, and then the prepared solution was heated at 90 °C overnight, resulting in a viscous gel. After that, the dried gel was heated at 400 °C for 2 h to prepare Nd-Fe-B precursors. The Nd-Fe-B oxide powders were annealed at 800 °C for 2 h to prepare the Nd-Fe-B oxide powders. Finally, the desired Nd_2_Fe_14_B nanoparticles were obtained under vacuum by the reduction–diffusion (R–D) process. That same year, Bhame et al. reported the preparation of Nd_2_Fe_14_B/α-Fe powders by the auto-combustion method [13]. The Nd-Fe-B oxide powders were obtained by glycine nitrate-based auto-combustion of mixed metal nitrates. Then, the oxides were subjected to the reduction–diffusion process with CaH_2_ to synthesize Nd-Fe-B magnetic powders. Subsequently, based on the auto-combustion method, the Nd_2_Fe_14_B nanoparticles were first successfully synthesized by Swaminathan et al. using the facile microwave-assisted combustion method in 2014 [14], and the reaction time was greatly saved. In 2014, Yu et al. proposed the preparation of Nd_2_Fe_14_B/*α*-Fe nanoparticles using thermal decomposition. The method included the conversion of metal acetyl acetone precursors to metal oxide via heating operation at a relatively low temperature of 300 °C [15]. Using a self-assembled block copolymer, the high-purity Nd_2_Fe_14_B nanoparticles were successfully synthesized by Yonekura and Wakayama in 2017 [16]. After stirring for 3 h at 90 °C, the prepared solution was calcined at 180 °C for 3 h and then at 350 °C for 6 h. The resulting precursors were oxidized at 800 °C for 6 h, and then the oxides were similarly reduced with CaH_2_. In 2018, Chaudhary et al. synthesized Cr alloyed Nd_2_(Fe, Co)_14_B magnetic nanoparticles with a grain size of 50~70 nm through the mechanochemical process [17]. Some low-cost raw materials (metal oxides) were first dried at 1000 °C for 10 h, mixed with the reducing agent calcium, and milled for 6 h. In the process of mechanical high-speed ball grinding, metal oxides are partially reduced and broken. Finally, the desired Nd_2_Fe_14_B nanoparticles were obtained. Almost all of these methods share the same three steps: synthesis of the Nd-Fe-B precursor or oxide(s); calcination of the precursor or oxide(s) to obtain Nd_2_Fe_14_B nanoparticles via the R–D method with reductants; and removal of by-products. The differences in preparation schemes lie mainly in precursor or oxide(s) preparation methods, and the subsequent reduction process is similar.

However, these techniques entail intricate procedures, extended durations, elevated temperatures, or specific equipment requirements for preparing Nd-Fe-B precursors. Hydrothermal synthesis of Nd_2_Fe_14_B particles has also been described by our group, allowing for simpler processes to produce Nd-Fe-B precursors at lower temperatures than most documented chemical approaches [18,19]. Moreover, the preparation procedure avoids using any organic solvents, promoting a physiologically and environmentally friendly approach. However, it is worth noting that the preparation process is typically time-consuming, requiring 4 to 6 h. On the other hand, the microwave hydrothermal approach has numerous advantages, primarily in terms of rapid and uniform heating of the reaction system, absence of temperature gradients, shortened reaction times, and the potential to accelerate chemical reactions in various reaction systems [20,21]. The utilization of microwave heating in hydrothermal processes is advantageous due to its ability to efficiently transport energy across the whole volume of the material, resulting in a reduced heating time. Additionally, since microwave heating operates on a molecular scale, the combination of microwave heating and hydrothermal techniques is anticipated to be effective in enhancing the quality of the oxide precursor. This, in turn, would lay the groundwork for the development of Nd-Fe-B particles with high coercivity. Due to the powerful penetrating ability of microwaves, which is sufficient for introducing large amounts of energy into the material, the material’s internal temperature increases rapidly. The microwave-assisted hydrothermal approach exhibits significant promise in various industrial applications due to its ability to reduce the impact of temperature gradients on the heating process [22]. As of now, the current work marks the first investigation into the production of Nd_2_Fe_14_B particles using the microwave-assisted hydrothermal technique.

The objective of this investigation is to provide an efficient and sustainable approach for synthesizing high magnetic Nd-Fe-B powders using a chemical process, potentially saving time and energy and promoting environmental friendliness.

## 2. Results and Discussion

### 2.1. Formation of a Nd-Fe-B Precursor

**Figure 1:**
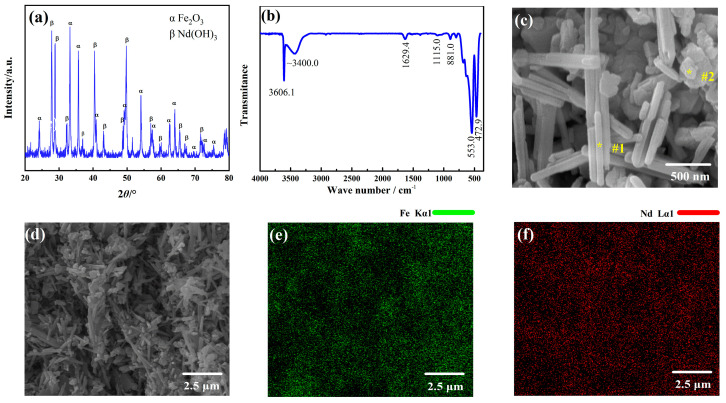
Characterization of as-prepared Nd-Fe-B precursor. (**a**) XRD pattern, (**b**) IR spectra, (**c**,**d**) SEM image, and elemental mapping of (**e**) Fe and (**f**) Nd of the Nd-Fe-B precursor.

The characterization of the as-prepared Nd-Fe-B precursor is illustrated in Figure 1. The phase composition of the precursor is investigated using XRD. The Nd-Fe-B precursors are shown to be composed of Fe_2_O_3_ and Nd(OH)_3_, as shown in Figure 1a. As the compound containing B is amorphous, no compounds containing B have been detected.

Figure 1b shows the IR spectra of the Nd-Fe-B precursors. The presence of a significant peak at 472.9 cm^−1^ can be attributed to the stretching vibrations of M (metal)-O bonds [23]. The peaks at 881.0 cm^−1^ and 1115.0 cm^−1^ are both assigned to the B-OH bending vibrations. These vibrations are indicative of the OH coordination compound of B. The bands at 1629.4 cm^−1^, which are indicative of the bending vibrations of water molecules, are attributed to the presence of Nd(OH)_3_ [24]. The peak at 553.0 cm^−1^ belongs to the signal of iron oxide. Sharp, intense bands at 3606.1 cm^−1^ and strong, broad bands at 3400.0 cm^−1^ are attributed to OH coordination compounds of metal ions and O-H stretching vibrations of the associated OH group [25], respectively. According to these findings, the precursor comprises OH coordination compounds of M and B and metal oxides. The XRD data indicates that the Nd-Fe-B precursor is a combination of Fe_2_O_3_ and Nd(OH)_3_. However, the absence of a compound containing B in Figure 1a renders it impossible to determine its formula.

The reactions of NaOH and H_3_BO_3_ can be represented as follows:

when NaOH is excess, H_3_BO_3_ + NaOH → Na[B(OH)_4_]
when NaOH is insufficient, H_3_BO_3_ + NaOH → Na_2_B_4_O_7_.

In the present instance, the solution exhibits a pH value of 9, leading to the hydrolysis of H_3_BO_3_ into B(OH)^4−^. Although sodium salt is water soluble, B-OH persists in the precursor after being washed. Hence, it can be inferred that the presence of B-OH may be attributed to the existence of large colloidal structures formed through the complexation of Nd(OH)_3_ gel with free B(OH)^4−^.

Therefore, the following Equation (1) can be employed to represent the processes involved in the fabrication of the Nd-Fe-B precursor:

Fe(NO_3_) 9H_2_O + Nd(NO_3_) 6H_2_O + H_3_BO_3_ + NaOH → Fe_2_O_3_ + Nd(OH)_3_ + Na[B(OH)_4_] + H_2_O (1)

In a recent study [26], it was shown that the precursor for Nd-Fe-B generated using the hydrothermal technique had an amorphous structure consisting of Nd(OH)_3_ gel, Fe(OH)_3_ gel, and a few large colloids containing B. However, microwaves contribute to the production of Fe_2_O_3_ and Nd(OH)_3_ crystals in the precursor. The improved crystallinity and quality of the final product can be attributed to the efficiency of microwaves in transmitting energy inside the material volume, operating at the molecular level.

Figure 1c depicts an SEM image of the Nd-Fe-B precursor powder, and demonstrates that these particles consisted of a significant quantity of columnar grains with a diameter of about 100 nm mixed with block-shaped particles. According to EDS analyses, the columnar particle located in the region indicated #1 (marked as “*”) is composed of 82.9 at% O and 17.1 at% Nd, respectively (see Figure S1, Supplementary Information). This composition suggests that the region includes a substantial quantity of Nd(OH)_3_ in comparison to Figure 1a. The block-shaped particles, denoted as #2 (marked as “*”), predominantly consisted of 31.8 at% Fe and 68.2 at% O (see Figure S2, Supplementary Information). The particles were identified as Fe_2_O_3_ grains. Figure 1e,f depict EDS maps illustrating the distribution of Fe and Nd inside the precursor powder. The results demonstrate a uniform and homogeneous dispersion of these elements, which is beneficial for the subsequent synthesis of Nd-Fe-B oxides. Boron, being a light element, was not detected in the mapping.

### 2.2. Nd-Fe-B Oxides

**Figure 2:**
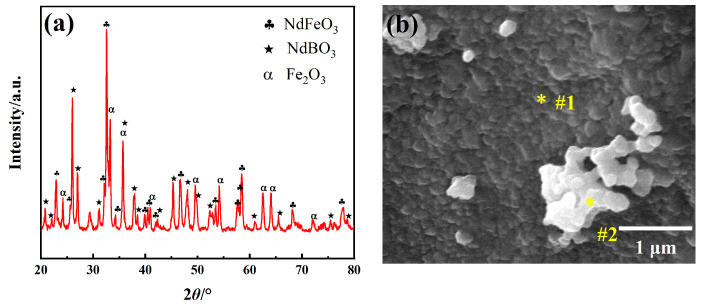
Characterization of as-prepared Nd-Fe-B oxides. (**a**) XRD pattern, (**b**) SEM image.

The as-synthesized Nd-Fe-B oxides are characterized and the results are shown in Figure 2. The X-ray diffractograms of Nd-Fe-B oxides prepared at 800 °C are shown in Figure 2a. As depicted in the figure, after the annealing process of the precursors, a composite oxide composed of NdFeO_3_, NdBO_3_ and Fe_2_O_3_ was generated. To determine the phase transition temperature and confirm the reaction detail during the oxidation process, the isochronal DSC/TG curves of the as-prepared Nd-Fe-B precursor from 100 to 800 °C are provided in Figure S3 (Supplementary Information) [27]. According to Figure S3a, the total weight loss was more than 25% of the whole precursor mass based on the TG curve. The DSC endothermic pick at about 260 °C was attributed to the decomposition of the hydrates. The exothermic peak at about 770 °C was associated with the phase transition from precursor to oxides, and there was only a slight change in weight during this process.

The samples were then calcined at 750 °C and 790 °C for 1 h, followed by XRD pattern analysis. The resulting XRD pattern is shown in Figure S3b,c. It can be seen that the sample was composed of Nd(OH)_3_, NdFeO_3_, Fe_2_O_3_, and B_2_O_3_ phases at 750 °C. At 790 °C, the sample consisted of NdFeO_3_, NdBO_3_, and Fe_2_O_3_. Thus, it can be inferred that NdFeO_3_ started to produce from Nd(OH)_3_ and Fe_2_O_3_ at about 750 °C. Meanwhile, the B(OH)^4−^ species undergoes thermal decomposition, resulting in the formation of B_2_O_3_. Then, the generated NdFeO_3_ immediately reacted with B_2_O_3_ to form NdBO_3_ at about 790 °C.

The chemical reactions associated with synthesizing Nd-Fe-B oxide can be expressed using Equations (2) and (3).

(2) $${2\mathrm{Nd}(\mathrm{OH})}_{3}{\;+\;\mathrm{Fe}}_{2}O_{3}\overset{\Delta }{\to }{2\mathrm{NdFeO}}_{3}{\;+\;3H}_{2}O$$

(3) $${2\mathrm{NdFeO}}_{3}{\;+\;B}_{2}O_{3}\overset{\Delta }{\to }{2\mathrm{NdBO}}_{3}{\;+\;\mathrm{Fe}}_{2}O_{3}$$

SEM was employed to characterize further the Nd-Fe-B oxide generated at 800 °C. According to Figure 2b, the structure of the Nd-Fe-B oxide consists of lamellar particles (region #1, marked as “*”) and equiaxed, agglomerated particles (region #2, marked as “*”), with particle diameters below 200 nm. The EDS scans show that the lamellar particle of the region marked #1 consisted of 24.3 at% Nd, 30.3 at% Fe, and 45.4 at% O (see Figure S4, Supplementary Information). The particles, as depicted in Figure 2a, are determined to be NdFeO_3_ grains. The equiaxed particle marked #2 comprises 39.5 at% Fe and 60.5 at% O, indicating that the area contains a large amount of Fe_2_O_3_ (see Figure S5, Supplementary Materials).

### 2.3. Reduction–Diffusion-Based Nd_2_Fe_14_B Phase Formation

**Figure 3:**
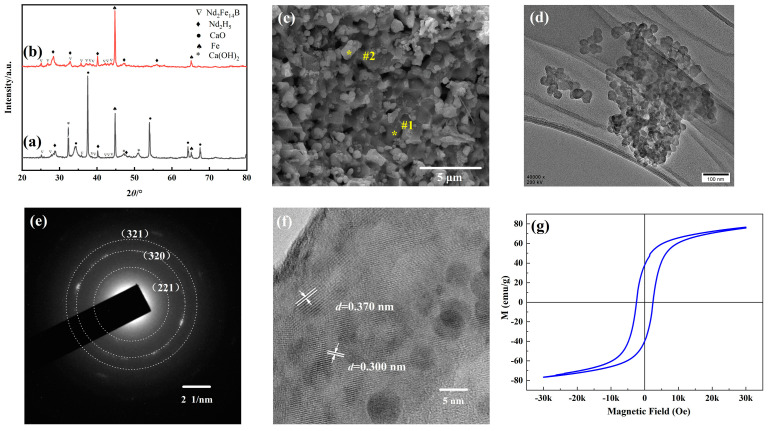
Characterization of as-prepared Nd−Fe−B magnetic particles. XRD pattern of the product (**a**) before washing and (**b**) after washing, (**c**) SEM image, (**d**) TEM micrograph, (**e**) SAED pattern, (**f**) HRTEM image, and (**g**) hysteresis loops at room temperature of Nd-Fe-B magnetic particles.

Following the reduction of oxides with CaH_2_, multiple phases, including Nd_2_H_5_, Nd_2_Fe_14_B, α-Fe, Ca(OH)_2_, and CaO, were identified in the resulting powder (see Figure 3a). After washing with ethanol and water, the final product obtained was a combination of Nd_2_H_5_, Nd_2_Fe_14_B, and α-Fe (see Figure 3b). To study the reaction process of R–D annealing, DSC/TG measurement of the mixture of Nd-Fe-B oxide and CaH_2_ powder was used to detect the sample’s phase transition temperature (See Figure S6a, Supplementary Materials). As can be seen, the TG result curve shows two distinct transitions between 100 °C and 800 °C, with a total 1.6% weight loss. The first weight loss was about 1.5% up to 420 °C in correspondence with an endothermic peak near 360 °C in the DSC curve. The second weight loss was 0.1% between 530 °C and 630 °C.

The samples were then subjected to R–D annealing at 365 °C, 425 °C, and 635 °C for 1 h, followed by XRD pattern analysis. The resulting XRD pattern is shown in Figure S6b–d (Supplementary Information); it can be seen that the sample was composed of NdFeO_3_, NdBO_3_, α-Fe, CaH_2_, and CaO phases at 365 °C. At 425 °C, a new phase Nd_2_H_5_ appeared, the amount of NdFeO_3_ and NdBO_3_ decreased while α-Fe increased. The sample consisted of Nd_2_H_5_, Nd_2_Fe_14_B, *α*-Fe, CaH_2_, CaO, and a little bit of remaining NdBO_3_ at 635 °C.

From the results for DSC/TG and XRD, we can infer the reaction process that occurred with R–D annealing. Firstly, the reduction of Fe_2_O_3_ with CaH_2_ occurred at about 365 °C, resulting in the formation of the α-Fe phase; between 365 °C and 425 °C, according to the change of phase composition, it indicates that NdFeO_3_ and NdBO_3_ were reduced by CaH_2_ to form the Nd_2_H_5_ and α-Fe phases during this period. However, no compounds containing B were detected, suggesting that the reduction product was B elemental. It is worth noting that B is an amorphous element. When obtained at lower temperatures, B is usually amorphous; only at high temperatures could it produce crystallization (pure B crystallizes only at up to 1800 °C) [28]). Since the synthesis temperature in this research was well below the crystallization temperature, B was amorphous and the XRD curves did not show the characteristic B peak. At 635 °C, the desired Nd_2_Fe_14_B phase was formed. Based on these observations, the following reaction mechanisms are proposed (Equations (4)–(7)):

(4) $${\mathrm{Fe}}_{2}O_{3}{\;+\;3\mathrm{CaH}}_{2}\overset{\Delta }{\to }{2\mathrm{Fe}+3\mathrm{CaO}+3H}_{2}↑$$

(5) $${4\mathrm{NdFeO}}_{3}{\;+\;12\mathrm{CaH}}_{2}\overset{\Delta }{\to }{2\mathrm{Nd}}_{2}H_{5}{+4\mathrm{Fe}+12\mathrm{CaO}+7H}_{2}↑$$

(6) $${4\mathrm{NdBO}}_{3}{\;+\;12\mathrm{CaH}}_{2}\overset{\Delta }{\to }{2\mathrm{Nd}}_{2}H_{5}{+4B+12\mathrm{CaO}+7\;H}_{2}↑$$

(7) $${2\mathrm{Nd}}_{2}H_{5}+28\mathrm{Fe}+2B\overset{\Delta }{\to }{\;2\mathrm{Nd}}_{2}{\mathrm{Fe}}_{14}{B+5H}_{2}↑$$

The Nd_2_Fe_14_B particles were washed and then characterized using FE-SEM, as illustrated in Figure 3c. The Figure 3c illustrates that the final product contained both clusters of dark particles (region #1, marked as “*”) and scattered light-colored particles (region #2, marked as “*”). EDS scans revealed that particles in region #1 were Nd_2_Fe_14_B, composed of 84.0 at% Fe and 16.0 at% Nd (see Figure S7, Supplementary Information). These particles displayed numerous pits and defects on their surface due to CaO shedding during the cleaning procedure. Particles in region #2 were primarily composed of Fe (94.9 at.%) and Nd (5.1 at.%) (Figure S8, Supplementary Information), indicating that Fe is the primary component in this area. The EDS analysis results of the final product are consistent with the XRD findings.

Figure 3d is a TEM image of the final product, which shows that the particles were equiaxial and monodisperse, with typical particle sizes of about 35 nm. The observed electron diffraction pattern in Figure 3e displays distinct ring patterns, indicating a polycrystalline structure of the product. The diffraction pattern in Figure 3e was analyzed, and the measured values of R from the outermost to the innermost regions were found to be 4.67, 4.08, and 3.33 1/nm, respectively. Consequently, the corresponding values of *d* were determined to be 0.214, 0.245, and 0.300 nm. These values correspond to the planes (321), (320), and (221) in the tetragonal crystal structure of Nd_2_Fe_14_B (JCPDS# 36-1296). Inter-fringe spacings of 0.300 and 0.370 nm are visible in the HRTEM image of a Nd_2_Fe_14_B particle (Figure 3f) and can be attributed to the (221) and (103) planes of Nd_2_Fe_14_B, respectively. The TEM findings are also in agreement with the XRD analysis.

The magnetic characteristics of Nd_2_Fe_14_B particles following the washing procedure have been illustrated in Figure 3g. The coercivity, remanence, and saturation magnetization of the as-synthesized powder were respectively determined to be 2.3 kOe, 37.5 emu/g, and 75.6 emu/g. Table S1 lists the properties of materials prepared by different chemical methods. The experimental results suggest that the magnetic powder synthesized in this study exhibits low magnetic properties, a common issue observed in the chemical synthesis of Nd_2_Fe_14_B magnetic powder [9,10,13,16,29,30,31,32]. Several factors contribute to this phenomenon. First, the cleaning process, aimed at removing by-products, introduces a significant number of surface defects on the magnetic powder. This, in turn, reduces the magnetocrystalline anisotropy and promotes the nucleation of reverse domains. Additionally, the cleaning process induces the product’s hydrogenation, leading to its magnetic characteristics’ degradation. Besides, small particles are easily oxidized. This is because small particles, especially in the nanometer range, have a higher surface area compared to their volume. This increased surface area makes them more reactive and prone to be oxidized. Furthermore, theoretically, as the magnetic nanoparticles have Nd_2_Fe_14_B and α-Fe biphases, the exchange coupling effect between the hard magnetic phase and the soft magnetic phase is conceivable, resulting in a higher coercivity. However, the maximum coercivity can be achieved only when the size of the soft phase is less than twice the domain wall thickness δ (~10 nm) of the hard phase [26]. In our study, the size of the resulting particle is about 35nm; because the size of the soft magnetic phase particles is larger than δ, the magnetic moment in the center of the particles cannot effectively interact with the hard magnetic phase because it is far away from the hard magnetic phase. Therefore, during demagnetization, the soft magnetic phase can easily generate the reverse magnetic domain nuclei and drive the hard magnetic phase to reverse, resulting in the reduction of the coercivity of the system [33]. In addition, the XRD result suggests that the product contained a lot of α-Fe, and the high content of α-Fe soft magnetic phase could cause the material to have a low coercivity. In summary, enhancing the performance of chemically synthesized Nd_2_Fe_14_B requires identifying a novel technique for by-product removal and optimizing particle size and percentage of Fe in the resulting product.

## 3. Experimental Section

### 3.1. Synthesis of Nd-Fe-B Precursors

The synthesis was performed utilizing Fe(NO_3_)_3_·9H_2_O (Alfa Aesar, Haverhillm, MA, USA, 99.9%), Nd(NO_3_)_3_·6H_2_O (Alfa Aesar, 99.9%), and H_3_BO_3_ (Alfa Aesar, 99.8%). To achieve the nominal composition of Nd_40_Fe_55_B_5_ (expressed by weight %), 5.26 g of Nd(NO_3_)_3_·6H_2_O, 16.16 g of Fe(NO_3_)_3_·9H_2_O, and 0.48 g of H_3_BO_3_ are dissolved in 50 mL of deionized (DI) water. The solution’s pH was adjusted to a value of 9 using NaOH (0.1 M). Before the microwave hydrothermal process, the solution was introduced into a 100 mL autoclave lined with Teflon. The reactor was then heated to 180 °C for 30 min in a microwave hydrothermal synthesizer. A multipurpose microwave chemical synthesizer (XH-800SE, Beijing Xianghu, China) was used as a microwave source.

The product precursors were allowed to cool naturally before being washed with ethanol and DI water, vacuum-filtered, and oven-dried.

### 3.2. Synthesis of Nd-Fe-B Oxides

Precursors were heated at a rate of 5 °C/min up to 800 °C and held at this temperature for 2 h in a tube furnace to produce Nd-Fe-B oxides. The oxide powders obtained were further exposed to a reduction–diffusion technique to develop the Nd_2_Fe_14_B powders.

### 3.3. Reduction-Diffusion Synthesis of Nd-Fe-B

In a glove box, the Nd-Fe-B oxides were combined with CaH_2_ (Sigma-Aldrich, St. Louis, MO, USA, 90–95%) at a weight ratio of 1:2. To execute the solid-state reaction and minimize the CaH_2_ oxidation effectively, the mixtures were compressed into wafer form under 30 MPa pressure for 3 min. Subsequently, the wafer was heated at 900 °C for 1.5 h in a tube furnace using a mixed flow of 95% Ar and 5% H_2_ gas. Subsequently, the wafers underwent a grinding process to transform them into fine powder within a glove box. The primary by-product, CaO, was separated using deionized water (DW) and ethanol. The remaining powders were then subjected to final drying in a vacuum oven.

**Figure 4:**
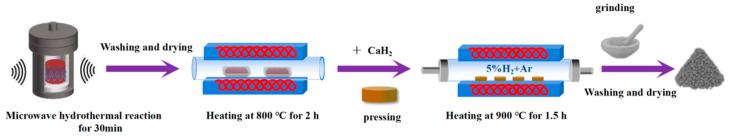
Schematic representation of Nd_2_Fe_14_B powders synthesis using microwave hydrothermal methods, followed by the R–D process.

The synthesized Nd_2_Fe_14_B particles were stored in a glove box due to their oxidation sensitivity. The synthetic procedure has been schematically depicted in Figure 4.

### 3.4. Characterization

The materials’ X-ray diffraction (XRD) patterns were acquired utilizing a Bruker DX2700B X-ray diffractometer (Bruker, Saarbrucken, Germany). The XRD measurements were conducted using Cu-Kα radiation, with a scanning rate set at 2°/min across the 2θ range of 20–80°. The infrared (IR) spectra were recorded on a Fourier transform infrared spectrometer (FTIR, Thermofisher, Waltham, MA, USA, IS50) using the KBr disc method. A Netzch DSC 449F3 DSC (Netzch, Selb, Germany) was used to detect the phase transition temperatures of the samples. The samples were analyzed for their microstructure and elemental composition utilizing a field emission scanning electron microscope (FE-SEM, Hitachi FE-4800 (Hitachi, Tokyo, Japan)) in conjunction with an EDS (Thermo System 7). Images were captured utilizing a high-resolution transmission electron microscope (HRTEM, JEOL 2010, 200 keV (JEOL, Tokyo, Japan)). The magnetic behavior at room temperature was analyzed using a physical property measurement system (PPMS, Quantum Design, San Diego, CA, USA), capable of applying up to 30 kOe of maximum magnetic field.

## 4. Conclusions

In conclusion, ultrafine Nd-Fe-B magnetic particles were developed using a microwave-assisted hydrothermal technique and a reduction-diffusion process. The use of microwave irradiation significantly reduces processing time. The synthesized Nd-Fe-B particles showed a coercivity of 2.3 kOe. The proposed approach can open up new opportunities for developing Nd_2_Fe_14_B/α-Fe nanocomposite magnets with exchange coupling and high energy density. Furthermore, the microwave-assisted combustion process, being time- and energy-efficient and environmentally friendly, is presumably not limited to the fabrication of Nd-Fe-B particles, but could be easily extended to the production of other rare earth-based magnetic materials.

## Data Availability

Data are contained within the article and supplementary materials.

## Supplementary Materials

The following supporting information can be downloaded at: https://www.mdpi.com/article/10.3390/molecules28237918/s1, Figure S1: EDS scans of selected gegions marked #1 in Figure 2; Figure S2. EDS scans of selected points marked #2 in Figure 2; Figure S3 Isochronal DSC of Nd-Fe-B precursor and XRD patterns of Nd-Fe-B precursor heated at different temperatures; Figure S4. EDS scans of selected points marked #1 in Figure 3; Figure S5. EDS scans of selected points marked #2 in Figure 3; Figure S6 Isochronal DSC of Nd-Fe-B oxide + CaH_2_ and XRD patterns of Nd-Fe-B oxide + CaH_2_ heated at different temperatures; Figure S7. EDS scans of selected points marked #1 in Figure 4; Figure S8. EDS scans of selected points marked #2 in Figure 4. Table S1 The comparison of preparation of Nd-Fe-B magnetic particles by different chemical methods [8,10,13,34,35,36].
