# Supplementary material for: Facile Synthesis of Nd2Fe14B Hard Magnetic Particles with Microwave-Assisted Hydrothermal Method
Source: Molecules. 2023 Dec 3;28(23):7918. doi: 10.3390/molecules28237918 (PMC10708516; doi:10.3390/molecules28237918)
Supplement: Supplementary file 1 [file molecules-28-07918-s001.zip › molecules-2678038-supplementary.pdf]

Supplementary Material for:

Facile Synthesis of $Nd_2Fe_{14}B$ Hard Magnetic Particles with Microwave-Assisted Hydrothermal Method

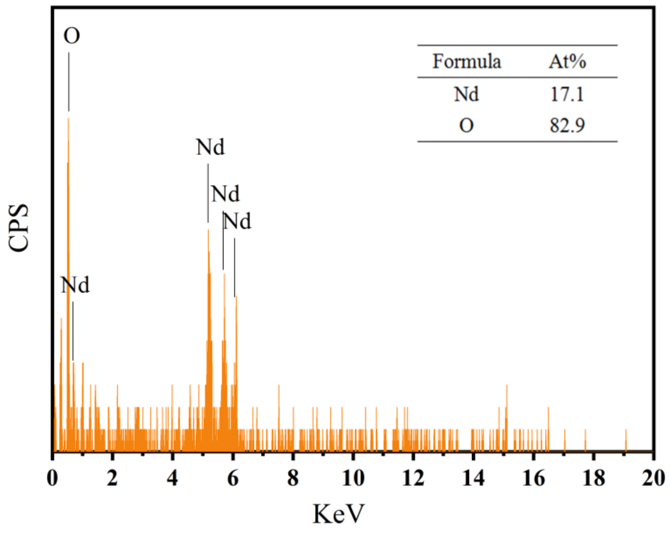

Figure S1. EDS scans of selected points marked #1 in Figure 2.

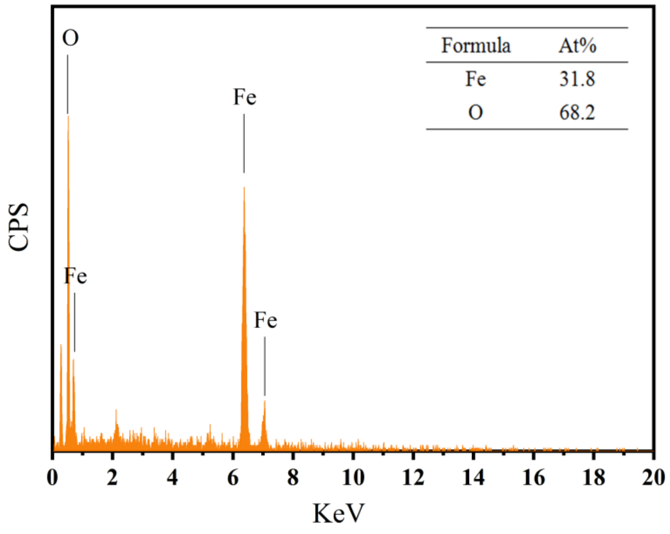

Figure S2. EDS scans of selected points marked #2 in Figure 2.

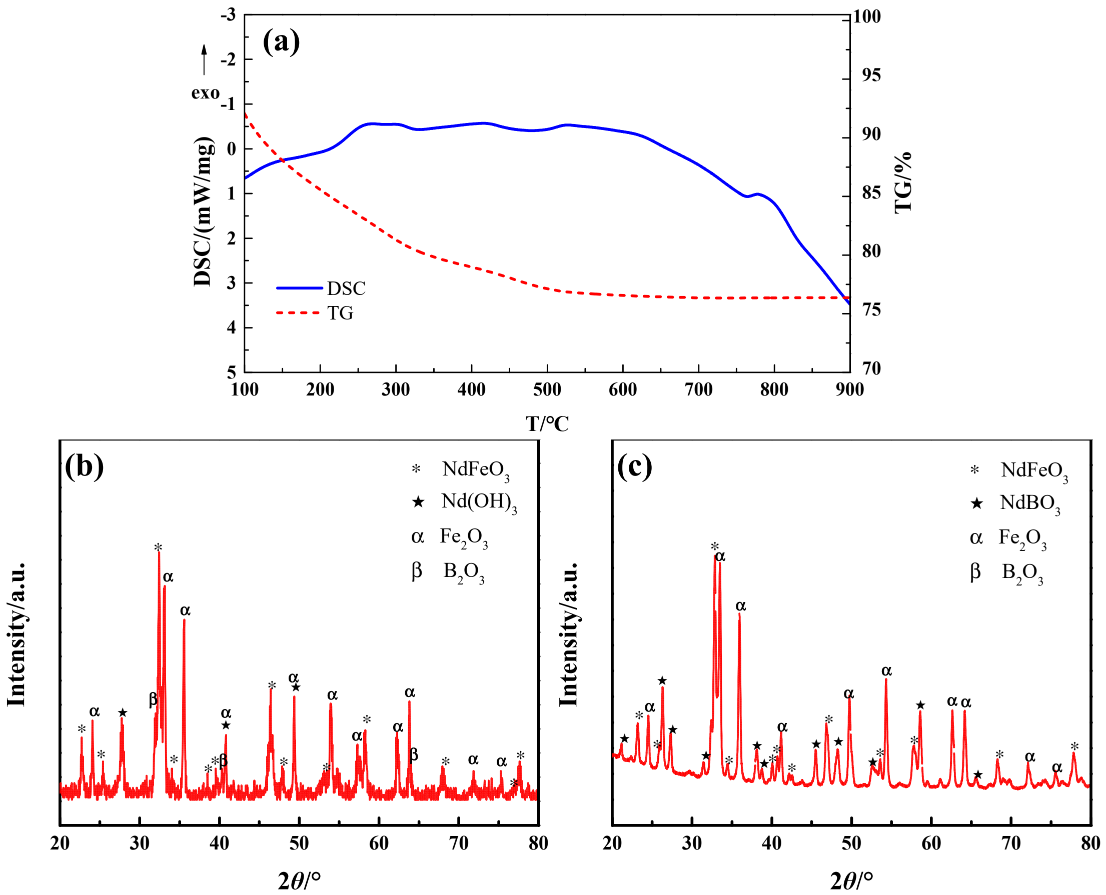

Figure S3 (a) Isochronal DSC of Nd–Fe–B precursor; XRD patterns of Nd–Fe–B precursor heated at temperatures (b) 750°C and (c) 790 °C.

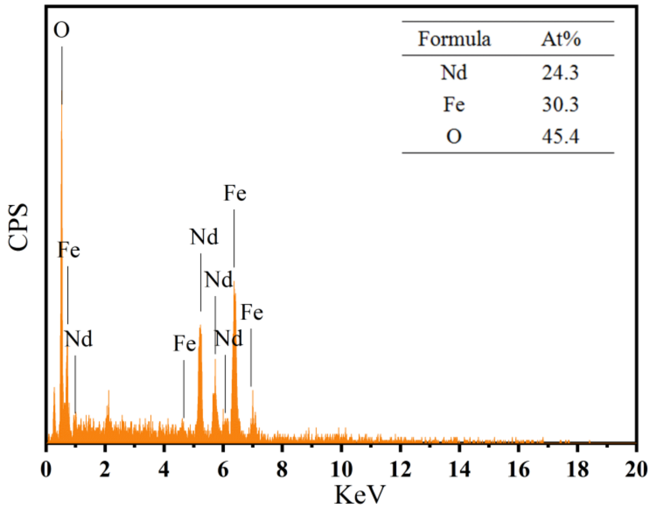

Figure S4. EDS scans of selected points marked #1 in Figure 3.

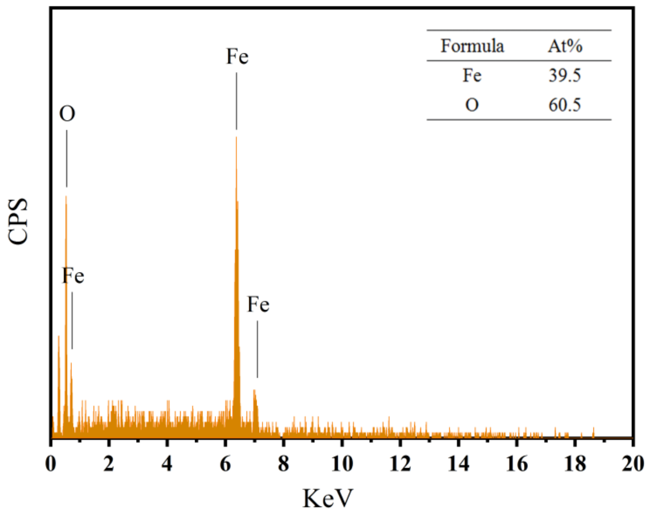

Figure S5. EDS scans of selected points marked #2 in Figure 3.

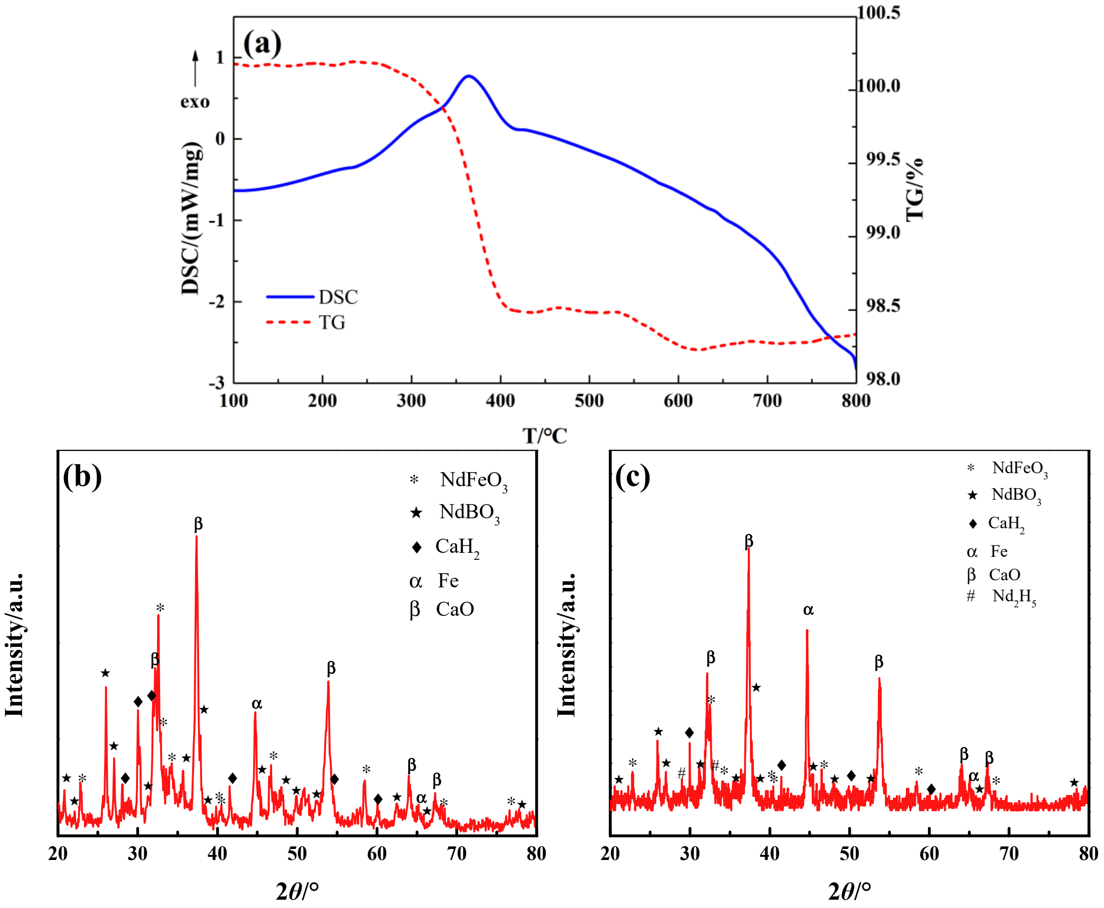

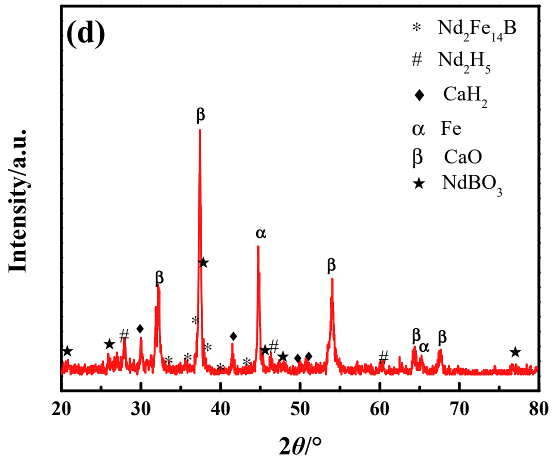

Figure S6 (a) Isochronal DSC of Nd–Fe–B oxide + CaH_2; XRD patterns of Nd–Fe–B oxide + CaH_2 heated at temperatures (b) 365°C , (c) 425°C and (d) 635 °C.

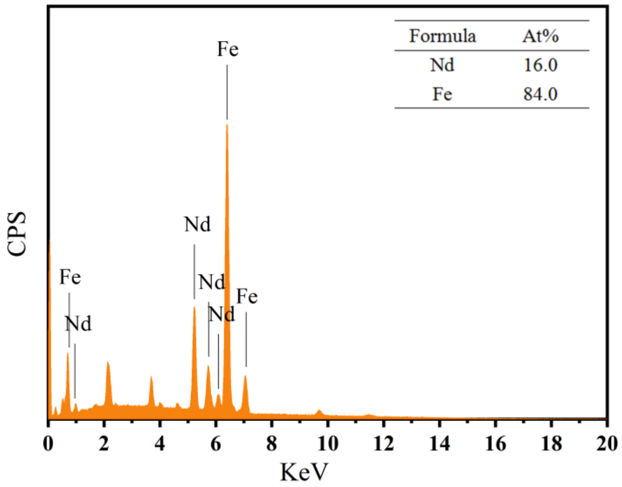

Figure S7. EDS scans of selected points marked #1 in Figure 4.

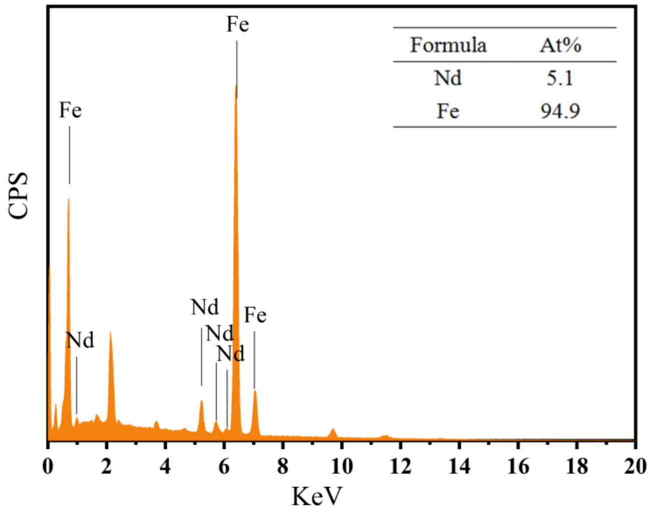

Figure S8. EDS scans of selected points marked #2 in Figure 4.

Table S1 The comparison of preparation of Nd-Fe-B magnetic particles by different chemical methods

Method	Composition	Ms (emu • g^{-1})	Mr (emu • g^{-1})	Hc (kOe)	Particle/Grain size(nm)	Ref.
Spray drying	$Nd_2Fe_{14}B$	148	135	3.9	-	[8]
Auto-combustion	$Nd_2Fe_{14}B$	80	69	3.3	44	[13]
Hydrothermal method	$Nd_2Fe_{14}B/\alpha$-Fe	107	25	1.2	~35	[34]
Sol-gel method	$Nd_2Fe_{14}B$	123	-	0.315	25	[10]
	$Nd_2Fe_{14}B/\alpha$-Fe	88.83	8.89	0.133	50-70	[35]
	$Nd_2Fe_{14}B$	14.17	-	1.02	-	[36]
Microwave-assisted Hydrothermal method	$Nd_2Fe_{14}B/\alpha$-Fe	75.6	36.7	2.3	35	This work
